# Editorial: Pathobiology, epidemiology and control of protozoan diseases of veterinary importance

**DOI:** 10.3389/fvets.2023.1235934

**Published:** 2023-06-30

**Authors:** Ragab M. Fereig, Ehab Mossaad, Charoonluk Jirapattharasate, Thu-Thuy Nguyen

**Affiliations:** ^1^Division of Internal Medicine, Department of Animal Medicine, Faculty of Veterinary Medicine, South Valley University, Qena, Egypt; ^2^Department of Pathology, Parasitology and Microbiology, College of Veterinary Medicine, Sudan University of Science and Technology, Khartoum, Sudan; ^3^Department of Pre-Clinic and Animal Science, Faculty of Veterinary Science, Mahidol University, Nakhon Pathom, Salaya, Thailand; ^4^Department of Pathobiology, School of Veterinary Medicine and Biomedical Sciences, Texas A&M University, College Station, TX, United States

**Keywords:** parasites, pathogenesis, vaccine, treatment, animals

Parasitic protozoan diseases represent a significant group of infectious illnesses threatening all animal species, including humans (1–4). These diseases include vector-borne diseases such as American trypanosomiasis (transmitted by the kissing bug), African trypanosomiasis and leishmaniasis (by flies), babesiosis and theileriosis (by ticks), and malaria (by mosquitoes). Such infections can lead to abnormal blood tests, severe symptoms and eventually, death (1, 2, 4). Other non-vector-borne protozoan diseases include toxoplasmosis, neosporosis, amoebiasis, coccidiosis, and cryptosporidiosis. These diseases are also dangerous, for example, toxoplasmosis and neosporosis inducing congenital transmission, abortion, fetal resorption, mummification, or the delivery of a fetus with congenital defects followed by death. Incidentally, neosporosis, amoebiasis, coccidiosis, and cryptosporidiosis are extremely contagious and widespread throughout the world in various animal species including ruminants, equines, and poultry (2, 3).

Even though protozoan infections have a significant impact on animal industry, public health, and global economy, most of them are frequently neglected in both low-income and high-income nations. Further, for the majority of protozoan parasites and their subsequent infections, there are currently no effective vaccinations or medications due to the complex life cycle and antigenic variation of the parasites, along with the emerging resistance and toxicity of current chemotherapeutic drugs (2, 3). Therefore, this Research Topic seeks to present the most recent information on three aspects: pathobiology, epidemiology, and control of protozoan diseases of veterinary and/or zoonotic interest. The goal of the current Research Topic was to centralize this knowledge in order to increase public awareness, as well as providing useful information for researchers and specialists on the prevention and control of protozoan diseases.

Seven published articles in this Research Topic are original studies covering the mentioned aspects on four infectious diseases: coccidiosis (da Silva et al.; Fang et al.), theileriosis (Elati et al.), neosporosis (Cao et al.; Gliga et al.; Metwally et al.; Zanet et al.), and toxoplasmosis (Metwally et al.).

Regarding coccidiosis, *Eimeria kongi* (*E. kongi*) causes coccidiosis in rabbits and was recognized as a new rabbit coccidia species. Driven by the currently limited information on *E. kongi*, Fang et al. investigated its pathogenicity, immunogenicity, endogenous development and drug sensitivity. In their study, Fang et al. discovered that *E. kongi* has a moderate pathogenicity in which the main clinical signs are diarrhea (20–60%) and weigh lost (100%) with targeted and colonization sites in the jejunum and ileum of the small intestine. Interestingly, while *E. kongi* lifecycle development is similar to *E. irresidua*, a highly pathogenic coccidia in domesticated rabbit, the development rate is faster; the cells are smaller; and the cell location is different. For treatment of *E. kongi* infections, sulfachloropyrazine sodium and decoquinate are most effective regarding the suppression of oocyte discharge (at 80.47 and 99.97% reduction) and increasing the body weight of the infected rabbits.

As the most significant disease in poultry, avian coccidiosis is highly endemic in northeastern Brazil (da Silva et al.). During an epidemiological surveillance of avian coccidiosis, 59% of the free-range chicken farms tested positive with *Eimeria* spp. From the positive chicken feces, eight species of *Eimeria* were identified. Among those identified, *E. necatrix* was the most prevalent. The study demonstrated that examination of oocyte morphology is a reliable and effective method that local veterinary laboratories can utilize as a routine diagnosis of avian coccidiosis. Additionally, the method presented is useful for epidemiological surveillance which will result in earlier treatment and better control of avian coccidiosis.

Tropical theileriosis is a protozoan disease caused by *Theileria annulata* (*T. annulata*). Currently, prevention of this disease is dependent on vaccination with attenuated *T. annulata* schizonts; however, the cultivation of *T. annulata* schizonts in media containing fetal bovine serum (FBS) has various drawbacks, such as its high manufacturing cost, inconsistent quality, and several ethical issues involved with its production. To address these drawbacks, Elati et al. attempted to establish serum-free culture conditions for *T. annulata*. The results of the study indicated that the freshly isolated parasites can be proliferated, frozen and thawed in serum-free media such as ISF-1. However, once the parasites are adapted to cultivation in the presence of FBS or resuscitated from frozen storage, propagation in serum-free media may not perform as well as cultivation in RPMI-FBS.

Another important protozoan disease, *Neospora caninum* (*N. caninum*) infection causes abortion in cattle globally. The control of the disease is primarily dependent on systematic serological testing of the animals and identification of the risk factors to prevent horizontal and vertical transmission of *N. caninum*. However, there are some aspects on the impact and control of this disease and the risk factors of transmission that are not fully understood. In line with that, the reports of Cao et al., Gliga et al., and Metwally et al. from their nationwide surveillance on *N. caninum* infections have culminated in an updated and useful information basis to fill that knowledge deficit. Metwally et al. detected high prevalence of *N. caninum* (24.6%) and *T. gondii* (5.3%) among cattle in the Beheira governorate. It should be noted that it is the first occurrence that both age and sex have been identified as risk factors of *N. caninum* transmission in Egypt. Next, in China, Cao et al. reported the seroprevalence of *N. caninum* infection at 13.6%. The study indicated that high prevalence is associated with large-scale farms and highly populated locations. Lastly, in Switzerland, Gliga et al. revealed the lowest seroprevalence of the three studies at 4.2%. One important risk factor determined by the study is the presence of rodents on the farm. Additionally, at the farm-level, rearing of replacement heifers and feeding of concentrated feed were considered as two protective factors. This result might be indicative to the success of the applied control policy for bovine neosporosis in Switzerland.

Lastly, *N. caninum* is not simply limited to domestic animals, but also affects wild animals. The study of Zanet et al. provides information on the incidence of *N. caninum* in wildlife animals in Italy and *N. caninum*'s congenital transmission in wild ungulates and carnivores. The transmission from parent to offspring was found at 87.5% among wild boars, foxes, and roe deer from five different provinces of Northwestern Italy. Further, infected pregnant mothers had a high risk for the development of the infection in the fetuses. According to this finding, the significance of this route in the parasite's maintenance within a sylvatic cycle warrant future investigation.

In conclusion, this Research Topic provides useful and insightful information on the basic and field research regarding protozoan parasites of veterinary and zoonotic importance. These findings represent potent solutions for understanding the biology and pathogenesis of the parasites, the current situation, prevalence, and risk factors of the infections and transmission. This information might be applicable for development of novel vaccines and efficient drugs for the control and prevention of such protozoan parasites.

## Author contributions

RF wrote the introduction and conclusion and supervised the work. EM and CJ wrote the comments and highlights to the articles. T-TN corrected the manuscript. All authors have contributed equally in preparation of this editorial.
